# Methicillin Resistant *Staphylococcus aureus* among HIV Infected Pediatric Patients in Northwest Ethiopia: Carriage Rates and Antibiotic Co-Resistance Profiles

**DOI:** 10.1371/journal.pone.0137254

**Published:** 2015-09-30

**Authors:** Martha Tibebu Lemma, Yohannes Zenebe, Begna Tulu, Daniel Mekonnen, Zewdie Mekonnen

**Affiliations:** 1 Department of Microbiology, Immunology and Parasitology, College of Medicine and Health Sciences, Bahir Dar University, Bahir Dar, Ethiopia; 2 Department of Biochemistry, College of Medicine and Health Sciences, Bahir Dar University, Bahir Dar, Ethiopia; 3 Biotechnology Research Institute, Bahir Dar University, Bahir Dar, Ethiopia; University of Iowa Carver College of Medicine, UNITED STATES

## Abstract

**Background:**

MRSA infections are becoming more prevalent throughout the HIV community. MRSA infections are a challenge to both physicians and patients due to limited choice of therapeutic options and increased cost of care.

**Objectives:**

This study was aimed to determine the prevalence of colonization and co-resistance patterns of MRSA species among HIV positive pediatric patients in the Amhara National Regional State, Northwest Ethiopia.

**Methods:**

Culture swabs were collected from the anterior nares, the skin and the perineum of 400 participants. *In vitro* antimicrobial susceptibility testing was done on Muller Hinton Agar by the Kirby-Bauer disk diffusion method, using 30 μg cefoxitin (OXOID, ENGLAND) according to the recommendations of the Clinical and Laboratory Standards Institute. Methicillin sensitivity/resistance was tested using cefoxitin. Data was analyzed by descriptive statistics and logistic regression model using Epi Info 7.

**Results:**

*S*. *aureus* was detected in 206 participants (51.5%). The prevalence of MRSA colonization in this study was 16.8%. Colonization by *S*. *aureus* was associated with male gender (OR = 0.5869; 95% CI: 0.3812–0.9036; p-value = 0.0155), history of antibiotic use over the previous 3 months (OR = 2.3126; 95% CI: 1.0707–4.9948; p-value = 0.0329) and having CD_4_ T-cell counts of more than 350 x 10^6^ cells / L (OR = 0.5739; 95% CI = 0.3343–0.9851; p-value = 0.0440). Colonization by MRSA was not associated with any one of the variables. Concomitant resistance of the MRSA to clindamycin, chloramphenicol, co-trimoxazole, ceftriaxone, erythromycin and tetracycline was 7.6%, 6%, 5.25%, 20.9%, 23.9% and 72.1%, respectively.

**Conclusion:**

High rates of colonization by pathogenic MRSA strains is observed among HIV positive pediatric patients in the Amhara National Regional state.

## Introduction

HIV-infected patients have increased *Staphylococcus aureus* colonization [1,2]. As colonization by Methicillin Resistant *S*. *aureus*(MRSA) is associated with increased risk of infection by MRSA [1,3,4], individuals at risk for both the colonization and infection by MRSA may serve as sources of outbreaks in both hospital and community settings.

Generally, MRSA colonization is said to occur in individuals who have frequent exposure to healthcare settings and in those with frequent antibiotic usage as well as immune suppression [1, 5–7]. Other factors listed in the literature as risk for health facility associated MRSA infection are age, duration and place of hospitalization, underlying disease, invasive procedures or devices, previous hospitalization, intensity of care, proximity to a MRSA-colonized patient, underlying dermatological diseases [8–10]. Male-to-male sexual intercourse and housing conditions are also cited as potential risk factors for community acquired MRSA (CA-MRSA) [11,12].

MRSA infections are a challenge for physicians in developing countries to treat because of the limited choice of therapeutic options available [13] and due to the possibility of concomitant drug resistance of the MRSA to other antimicrobials. MRSA are also a challenge to patients in developing settings due to increased cost of care [14, 15]. The financial burden of MRSA care in regions of limited resource, such as Ethiopia, is not expected to be easy because there are other priorities such as TB, HIV and malaria already [16].

Infection prevention and control measures have been the primary means to attempt to limit the spread of MRSA. Management of MRSA infections among HIV infected children in the study area generally tends to be empirical based on reports from literature. To our knowledge, there are no reports on colonization rates and antibiotic susceptibility patterns of MRSA among HIV infected patients in the study area and Ethiopia in general. This study is aimed to provide an overview of the epidemiology and microbiology of MRSA in the Amhara National Regional State (ANRS) with special focus on HIV positive pediatric patients.

## Patients and Methods

### Study Design

A total of 400 study participants were recruited from the Pediatric HIV clinics of FelegeHiwot Referral Hospital, Dessie Referral hospital and Debre-Tabor Referral Hospital, in the Amhara National Regional State. Eligible participants were HIV-infected, under 15 years of age, receiving medical care at the aforementioned health facilities. Patients who were on antibiotic treatment for any bacterial infection during the time of data collection were excluded from the study. Any voluntary participants who visited the clinics from December 2013 through April 2014 were invited to participate in the study.

### Variables

A structured questionnaire was used to collect microbiologic data (such as colonization by *S*.*aureus* and antibiotic suceptibility profiles) as well as pertinent data from patient records (such as demographic characteristics, immune status, type of ARV drugs used for therapy, history of antimicrobial drug use within the past 3 months, living condition, and history of skin infections).

### Laboratory Procedures

From each participant, specimens for *S*. *aureus* culture were collected from the anterior nares, the skin of the back and the perineum using sterile broth moistened swabs.

The swabs were aseptically inoculated onto Sheep Blood Agar (SBA) and Mannitol Salt Agar (MSA) (Becton Dickinson)media. The culture plates were examined after 24–48 hours of incubation at 35°C (under anaerobic conditions for SBA and aerobic conditions for MSA plates). Sub culturing was done on both SBA and MSA.

Isolates were confirmed using Gram stain. Colonies with Gram positive cocci isolates were further tested for catalase and coagulase activity. *In vitro* antimicrobial susceptibility testing was done on Muller Hinton Agar by the Kirby-Bauer disk diffusion method using chloramphenicol (30ug), ceftriaxone (30 ug), ciprofloxacin (5 ug), clindamycin (10ug), erythromycin (15ug), tetracycline (30 ug) and trimethoprim-sulfamethoxazole (25ug).

Clinical and Laboratory Standards Institute (CLSI) guidelines were used for interpretation of zones of inhibition. Methicillin sensitivity/resistance was tested using 30 μg cefoxitin (OXOID, ENGLAND). The methicillin-susceptible strain of *S*. *aureus* ATCC 29213 and the MRSA isolate BMB9393 were used as controls.

### Definitions

At each study visit, participants were classified as MRSA colonized if MRSA was detected from any one of the specimen collection sites. Participants were classified as colonized with methicillin-susceptible *S*. *aureus* (MSSA) if MSSA was detected and MRSA was not detected. Participants colonized with both MSSA and MRSA were classified as MRSA colonized.

### Statistical Methods

The primary analysis compared participants in whom a MRSA colonization had occurred with those without MRSA colonization. All analyses were performed using Epi Info 7.

By using a binary logistic regression model, adjusted Odds ratios (AOR) and 95% Confidence Intervals were calculated to identify variables associated independently with the development of MRSA colonization. All variables with the p value less than zero point two (p<0.2) in univariate analysis were included in a multivariate model. Statistical significance was indicated by a p value <0.05.

### Ethics Statement

Ethical clearance was obtained from the Institutional Review Boards (IRB) of the Biotechnology Research Institute (BRI) of Bahir Dar University. Verbal consent was obtained from the participants' guardians in order to avoid cultural issues that would have been raised by signing documents amongst the highly stigmatized HIV patients in the study area. Additionally, an affirmation of a desire to give specimen was secured from older participants (7–15 years of age). A script of the oral consent written in Amharic language, which was approved by the IRBs, was used as a guide to obtaining the verbal consent. A log was prepared to document responses to the invitations to participate in the study. Permission to conduct the study was obtained from the Amhara National Regional Health Bureau and the medical directors’ offices of the respective hospitals included in the study.

## Results

From the total 400 HIV-infected pediatric patients, 1200 specimens were collected. Most participants (77%) were urban dwellers, with the majority having a follow up at the Felege Hiwot Referral Hospital in Bahir Dar city (47%), followed by Dessie Referral Hospital in Dessie city (37.5%) and the rest from Debre-tabor Referral Hospita in Debre-tabor city (15.5%).

Males accounted for 56% of the cases (Male: Female ratio = 1.3) with median age of 10 (IQR = 4). The majority of participants (37%) had a base line WHO stage III HIV diagnosis. The median most recent CD4 cell count was 597 cells/μL. Antiretroviral therapy had been taken by 79.8% of the patients, with the majority (36%) on Zidovudine based standard regimen. Nearly 80% of the patients were on Co-trimoxazole prophylaxis therapy (CPT). About 2.5% of the patients had history of treatment failure while 3.5% had history of hospital admission within the past 1 year. The demographic and clinical parameters of the participants is summarized on Table 1.

**Table 1:** Baseline demographic data in HIV-infected children who gave skin, nasal and perineal samples, Amhara National Regional State, 2014.

Variable	Patients, n (%)
Female	176 (44)
Type of residence, n (%)	
Urban	397 (99.2)
Rural	3 (0.8)
Median age (IQ range)	10 (4)
WHO Stage, n(%)	
I	93 (23.2)
II	139 (34.8)
III	149 (37.2)
IV	19 (4.8)
CD4 count, Median (IQR)	
Baseline	322 (392)
Most recent	597 (493)
Hospitalization within the past 12 months, n (%)	
Yes	14 (3.5)
No	386 (96.5)
History of antibiotic use in the past 3 months, n(%)	
Yes	33 (8.2)
No	367 (91.8)
History of treatment with HAART, n (%)	
Taking HAART	318 (79.5)
Not on ARVs	82 (20.5)
Months on HAART, Mean	36.24
History of ARV treatment failure, n (%)	
Yes	10 (2.5)
No	390 (97.5)
Co-trimoxazole Preventive Therapy, n (%)	
Yes	318 (79.5)
No	82 (20.5)
Months on CPT, Mean	34.91

Overall, 281 of the specimens (23.4%) contained *S*. *aureus*, of which 73 (26%) were identified to be MRSA isolates. These isolates were identified from any of the three specimen collection sites of 206 participants (51.5%). Out of these 206 participants with *S*. *aureus* colonization, 67 of them (32.5%) were colonized by MRSA. Thus, the prevalence of MRSA colonization in this study was calculated to be 16.8% (i.e., 67 out of 400 total participants).

**Fig 1:**
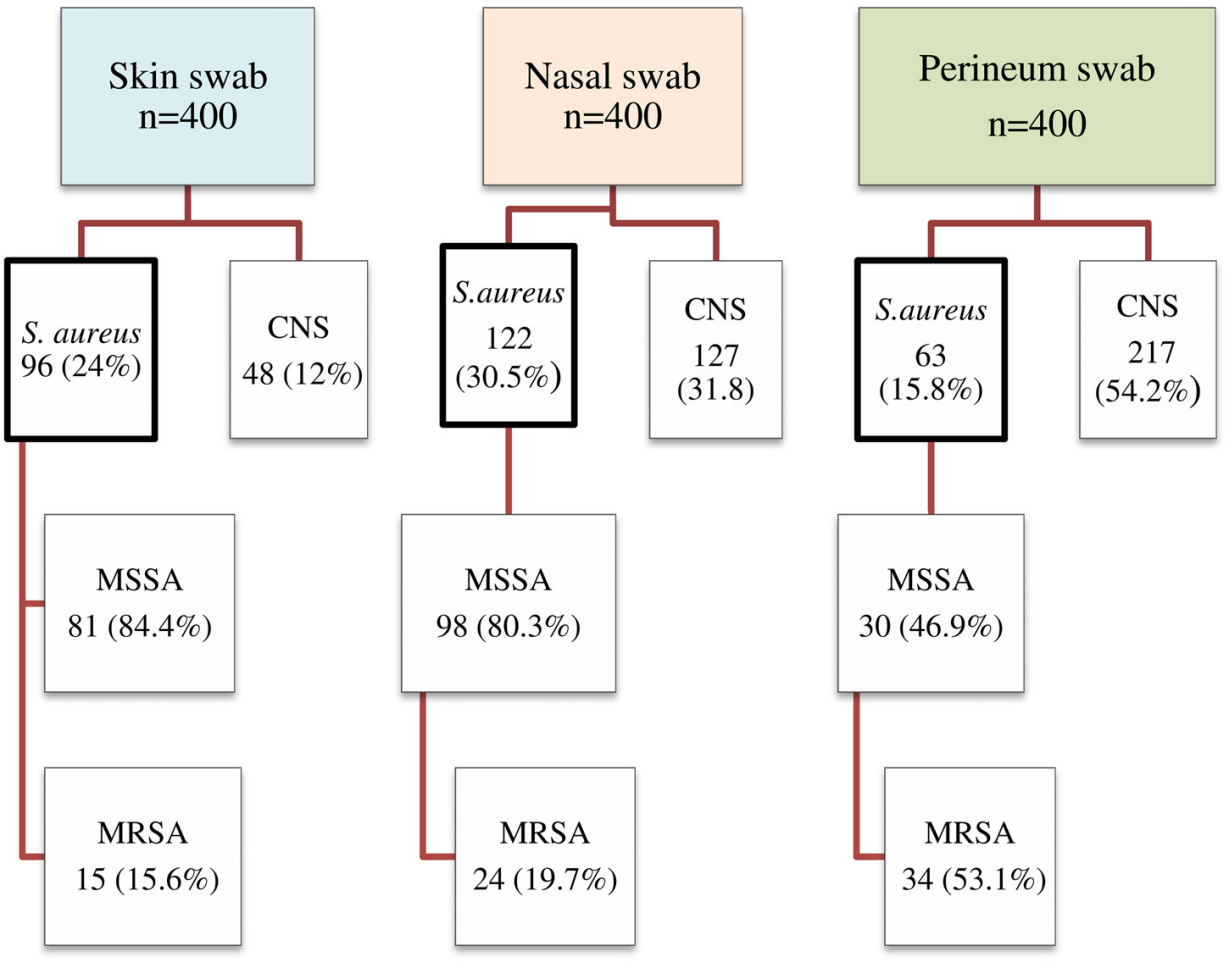
Numbers of *S*. *aureus* clinical isolates by type of specimen among 400 HIV-infected children, Amhara National Regional State, 2014.

The majority of the MRSA species (47.5%) were isolated from the perineum specimens, followed by nasal (32.8%) and skin specimens (19.7%) (Fig 1). In addition, MRSA colonization at two distinct specimen collection sites was detected in 1.25% of the participants. However, no patient had reports of MRSA colonization at all three sites simultaneously.

Additionally, MSSA was detected in 209 (17.4%) patient samples, while coagulase negative staphylococci (CNS) were identified from 392 (32.7%) of the patient samples.

In logistic regression analysis (Tables 2 and 3), there were no significant associations between MRSA colonization and the independent variables. However, colonization by *S*. *aureus* was observed to have a statistically significant association with each of male gender (OR = 0.5869; 95% CI: 0.3812–0.9036; p-value = 0.0155), history of antibiotic use over the previous 3 months (OR = 2.3126; 95% CI: 1.0707–4.9948; p-value = 0.0329) and having CD_4_ T-cell counts of more than 350 x 10^6^ cells / L (OR = 0.5739; 95% CI = 0.3343–0.9851; p-value = 0.0440).

**Table 2:** Correlation between Variables and Colonization by *S*. *aureus* among 400 HIV-Infected Pediatric Patients, Amhara National Regional State, 2014.

Covariate	Odds ratio	p-value	95% CI
Gender (Male/Female)	0.5869	0.0155	0.3812–0.9036
Age Groups	0.9364	0.858	0.4557–1.9242
History of hospitalization in the past 3 months	0.7334	0.6398	0.2002–2.6871
History of antibiotic therapy in the previous 3 months (yes/No)	2.3126	0.0329	1.0707–4.9948
Initiation of ART	0.818	0.5594	0.4167–1.6059
Type of HAART regimen	0.9062	0.0928	0.8078–1.0165
Duration on HAART	1.2616	0.069	0.9821–1.6208
Initiation of CPT	1.3286	0.4248	0.6612–2.6695
Duration on CPT	1.3377	0.167	0.8854–2.0210
History of ART failure	0.6613	0.5425	0.1748–2.5024
Baseline WHO stage	1.0798	0.5545	0.8370–1.3931
Most recent WHO stage	0.9988	0.9845	0.8855–1.1266
Baseline CD_4_Category*	0.7745	0.2294	0.5106–1.1749
Most Recent CD_4_Category*	0.5739	0.044	0.3343–0.9851

*CD_4_T-cell count of 350 x 10^6^ cells / L or more per ml versus CD_4_T-cell counts under 350 x 10^6^ cells / L

**Table 3:** Correlation between Variables and Colonization by MRSA among 400 HIV-Infected Pediatric Patients, Amhara National Regional State, 2014.

Covariate	Odds ratio	p-value	95% CI
Gender (Male/Female)	0.9772	0.9324	0.5733–1.6657
Age Groups	1.0104	0.9615	0.6633–1.5392
History of hospitalization in the past 3 months	1.0297	0.9704	0.2193–4.8352
History of antibiotic therapy in the previous 3 months (yes/No)	2.608	0.027	0.1010–2.5420
Initiation of ART	1.0167	0.9696	0.4346–2.3786
Type of HAART regimen	0.9679	0.6562	0.8383–1.1175
Duration on HAART	0.9472	0.7921	0.6329–1.4176
Initiation of CPT	0.9714	0.93	0.5090–1.8541
Duration on CPT	1.0226	0.9367	0.5886–1.7767
History of ART failure	0.394	0.2076	0.0926–1.6771
Baseline WHO stage	0.863	0.4037	0.6107–1.2195
Most recent WHO stage	1.0877	0.305	0.9263–1.2771
Baseline CD_4_ Category*	0.8436	0.5508	0.4825–1.4751
Most Recent CD_4_Category*	0.6809	0.2547	0.3514–1.3193

*CD_4_T-cell count of 350 x 10^6^ cells / L or more per ml versus CD_4_T-cell counts under 350 x 10^6^ cells / L

In toto, as much as 20.9% of the MRSA isolates were resistant to ceftriaxone.Resistance to each of ciprofloxacin or erythromycin was noticed in 23.9% of the isolates.

While the figure for tetracycline resistance was highest (72.1%), clindamycin, chloramphenicol and co-trimoxazole had resistance rates of 7.6%, 6% and 5.25% respectively. Table 4 shows the antibiotic susceptibility pattern of the MRSA isolates to common antimicrobial agents in the study area.

**Table 4:** Antibiotic co-resistance pattern of MRSA isolated from HIV positive pediatric patients, Amhara National Regional state, 2014.

Antibiotic	Per cent	95% CI
Chloramphenicol	6	0.32–2.72
Ceftriaxone	20.9	2.00–5.94
Ciprofloxacin	23.9	2.38–6.55
Clindamycin	7.6	0.46–3.06
Co-trimoxazole	5.25	3.36–8.04
Erythromycin	23.9	2.38–6.55
Tetracycline	72.1	9.28–15.96

## Discussion

This study is the first of its kind to assess rates of *S*. *aureus* colonization of HIV infected pediatric patients in the study area particularly and Ethiopia at large. The findings underscore the existence of alarming rates of MRSA colonizers that are simultaneously resistant to some commonly prescribed antimicrobial agents.

Overall, the rate of *S*. *aureus* colonization reported from this study (51.5%) was higher than that of a study involving HIV positive children in Durban, South Africa (26.5%) [17].

More *S*. *aureus* were identified from the nasal specimen than the perineal swabs, at rates of approximately half of the values for nasal carriage. Although intestinal carriage rates of *S*. *aureus* among HIV infected children have not been widely investigated, studies involving different sets of adult population generally indicate comparatively higher figures of nasopharyngeal carriage than those of perineal carriage [18–20].

Our finding on the prevalence rate of nasopharyngeal colonization by *S*. *aureus* from this study was consistent with specific figures from West Bengal (24%) [21] and Cambodia (30.4%) [22]. However, higher rates were observed among children with HIV-1 infection in a hospital-based cross-sectional study from Brazil (45.16% vs. 19%) [23]. While these differences could be attributed to differing use of antimicrobials in the corresponding study areas, all these figures are generally indicative of requiring high attention.

The proportion of MRSA identified in this study was compared with reports from two different studies conducted in Soweto (South Africa) (39% and 60%) [24,25]. A small retrospective case-control study undertaken among patients followed at the Texas Children's Hospital Retrovirology Clinic indicated even higher rates of MRSA (82%) [26].

In our study, colonization by *S*. *aureus* had a significant statistical association with some variables. Nonetheless, we observed differing levels of consistency with findings of previous studies. For example, history of antibiotic use over the previous 3 months increased the likelihood of colonization by Staphylococci (OR = 2.3126; 95% CI: 1.0707–4.9948; p-value = 0.0329), while high CD_4_ T-cell counts were found to have got a protective effect. In contrary, Bhattacharya *et al* reported no association with immune status or recent antibiotic use [21]. Also, male gender had lower odds for colonization by *S*. *aureus* (OR = 0.5869; 95% CI: 0.3812–0.9036; p-value = 0.0155), while Bogaert *et al* indicated the opposite amongst healthy children (OR 1.46, 1.25–1.70) [27]. Other reports had found associations between age and *S*. *aureus* carriage in both healthy as well as HIV infected children, though there are inconsistencies with the specific age groups for the peak incidence for the colonization by *S*. *aureus* [27, 28]. No statistical relationship was observed with age in this study.

The assessment of the antibiotic susceptibility profiles of the MRSA isolates generally indicated high rates of co-resistance of MRSA to commonly prescribed antibiotics such as ceftriaxone, erythromycin and tetracycline. Groom *et al* [24] had similarly reported high figures of antibiotic resistance of MRSA to tetracyclines and erythromycin, while their findings on TMP—SMX and clindamycin co-resistance were much higher than our observation (94% and 65% respectively). Clindamycin resistance was observed in 19% of the MRSA isolates [29] in a different study.

The observed low rates of resistance to clindamycin, chloramphenicol and co-trimoxazole might suggest that most of these colonizations were caused by CA-MRSA rather than HA-MRSA strains [30–33]. Strain typing of the MRSA clinical isolates by molecular tools is recommended. Nevertheless, the high rates of concomitant drug resistance to the commonly available reserve intravenous antibiotics for use among the pediatric population needs to be given a special attention. This is because *S*. *aureus* is among the most common pathogens encountered in pediatric practice [34]. Additionally, reports of epidemics due to MRSA are not uncommon [34]. As all of the participants of this study were ambulatory, those that are colonized by the MRSA could easily transmit the pathogenic bacteria to other members of the community.

Staphylococcal infections tend to be serious necessitating aggressive treatment with specific and effective antimicrobial agents. Therefore, colonization by multidrug resistant MRSA of immunocompromised patients warrants intervention.

The authors of this study know, from anecdotal information, that intravenous ceftriaxone is one of the antibiotics recommended for the treatment of Staph infections in the study area. However, in the era of growing resistance to cephalosporins, cheaper alternatives need to be available. The cost of vancomycin and intravenous clindamycin is well above the affordability range of poor HIV patients and families, who need to meet the cost of other demands such as that of nutrition and treatment for opportunistic infections.

Based on the findings of this study, we recommend the use of chloramphenicol, co-trimoxazole or clindamycin for the management of MRSA infections among HIV infected patients in the ANRS. Additionally, decolonization and prompt institution of MRSA surveillance need to be considered by policy makers as these individuals might act as sources of outbreak.

One limitation of this study is its design in that it is a cross sectional study that does not allow comparison with the MRSA colonization rate of HIV negative children. Additionally, it was difficult to determine whether the colonization by *S*. *aureus* was persistent or intermittent. Lack of incorporation of viral load results was another limitation which would have made checking statistical associations between level of immune suppression of participants and colonization by MRSA more objective.

## Conclusion

High rates of colonization by pathogenic MRSA strains was observed among HIV positive pediatric patients in the Amhara National Regional state. The MRSA isolates from colonizers of skin, nose or perineum of HIV-infected children were found to be concomitantly antibiotic resistant to cephalosporin, tetracycline and erythromycin.

## Recommendation

We recommend antibiotics appropriate for multidrug resistant MRSA infections be introduced to the ANRS. There is an urgent need to further document the spectrum and antibiotic resistance profiles of HIV-infected children in under-resourced communities. Molecular analysis of the methicillin resistant *S*. *aureus* bacterial isolates for strain typing is planned as a continuation of this study.
